# Effects of mechanical stretching on the morphology of extracellular polymers and the mRNA expression of collagens and small leucine-rich repeat proteoglycans in vaginal fibroblasts from women with pelvic organ prolapse

**DOI:** 10.1371/journal.pone.0193456

**Published:** 2018-04-09

**Authors:** Sumei Wang, Dongyuan Lü, Zhenyu Zhang, Xingyuan Jia, Lei Yang

**Affiliations:** 1 Department of Obstetrics and Gynecology, Beijing Chaoyang Hospital, Capital Medical University, Beijing, China; 2 Center for Biomechanics and Bioengineering, Key Laboratory of Microgravity (National Microgravity Laboratory) and Beijing Key Laboratory of Engineered Construction and Mechanobiology, Institute of Mechanics, Chinese Academy of Sciences, Beijing, China; 3 Medical Research Center, Beijing Chaoyang Hospital, Capital Medical University, Beijing, China; University of Insubria, ITALY

## Abstract

To determine the effect of mechanical stretching load and the efficacy of postmenopausal estrogen therapy (ET) on pelvic organ prolapse (POP), vaginal fibroblasts isolated from postmenopausal women with or without POP were subjected to 0.1-Hz uniaxial cyclic mechanical stretching (CS) with 10% elongation and 10^−8^ M 17-β-estradiol (E_2_) treatment. We investigated the morphological characteristics of extracellular polymers using scanning electron microscopy (SEM) and monitored the mRNA expression of type I collagen (COL I) and type III collagen (COL III) as well as the small leucine-rich proteoglycan (SLRP) family members decorin (DCN), biglycan (BGN), fibromodulin (FMO), and lumican (LUM), using real-time quantitative polymerase chain reaction (RT-PCR). Using SEM, certain viscoelastic polymers were found to be randomly distributed among fibroblasts, which for normal fibroblasts formed clusters of plum flower-like patterns under static-culture conditions and resembled stretched strips when stretched in culture, whereas polymers among POP fibroblasts resembled stretched strips under static-cultured conditions and presented broken networks when stretched in culture. RT-PCR revealed that COL I, DCN, BGN, FMO, and LUM mRNA expression was significantly higher in POP than in normal fibroblasts under static-culture condition. Following CS, COL I and BGN mRNA expression was significantly up-regulated in normal fibroblasts, and DCN and FMO mRNA expression was down-regulated in POP fibroblasts. Following concomitant CS and E_2_ treatment, significantly elevated COL I and DCN mRNA expression was observed in normal fibroblasts, and significantly elevated COL I and BGN mRNA expression was observed in POP fibroblasts. COL III mRNA expression was not significantly different between the POP and normal group, and CS did not significantly affect expression in either group, though COL III was down-regulated in normal fibroblasts concomitantly treated with E_2_ and CS. We conclude that the morphological distribution of extracellular polymers in POP fibroblasts exhibited higher sensitivity and lower tolerance to stretching loads than do normal fibroblasts. These mechanical properties were further reflected in the transcription of COL I. Defects in the compensatory function of BGN for DCN and LUM for FMO exist in POP fibroblasts, which further affect the structure and function of COL I in response to stretching load, ultimately resulting in abnormal reconstruction of pelvic supportive connective tissues and the occurrence of POP. ET can maintain stretching-induced elevations in COL I and DCN transcription in healthy women and improve stretching-induced COL I, DCN, BGN, and FMO transcriptional changes in POP women to prevent and improve POP. Only down-regulated COL III transcription was observed upon concomitant CS and E2 treatment in normal fibroblasts, which suggests that the tensile strength, not the elasticity, of the supportive connective tissues is damaged in POP and that the higher tensile strength induced by ET in healthy fibroblasts prevents POP. These findings confirm the role of higher sensitivity and lower tolerance to mechanical stretching in the pathogenesis of POP and further provide evidence supporting the use of ET to prevent and inhibit POP in postmenopausal women.

## Introduction

Pelvic organ prolapse (POP) is the abnormal protrusion of pelvic organs into the vaginal canal or beyond the vaginal opening, which may cause a series of symptoms in the urinary, genital, and bowel tracts that adversely affect the quality of life of affected individuals. Despite being a common disease affecting 41.1% of women aged 50–79 years [1], the exact etiology and pathogenesis of POP remain poorly understood. Many risk factors related to POP, including vaginal delivery, advanced age, menopause, estrogen deficiency, chronic cough, obesity, constipation, and heavy lifting [2–4], may cause abnormal metabolism and remodeling of the pelvic supportive connective tissues, thereby affecting the mechanical properties of these tissues and resulting in the occurrence and progression of POP.

In the supportive system of the pelvic floor, fibrous connective tissues surrounding the pelvic organs form fascia and ligaments to provide mechanical strength to support the vagina and its adjacent organs. Meanwhile, due to their specific anatomical location, these tissues are subjected to constant mechanical tensile loading from abdominal pressure and gravity [5–7]. The fascia and ligaments of the pelvic floor mainly comprise dense connective tissues containing fibroblasts and extracellular matrix (ECM) secreted by fibroblasts. In addition to providing a microenvironment for cells to shape tissue-specific function, the ECM provides mechanical strength and support for tissue. Thus, any hypothesis seeking to explain the etiology and pathogenesis of POP must address the remodeling of fibroblasts and ECM. In a previous study concerning the effects of a 0.1-Hz uniaxial cyclic mechanical stretching (CS) load with 10% elongation on the morphology and cytoskeleton of vaginal fibroblasts in vitro, we confirmed the mechanosensitivity of these fibroblasts and verified that POP fibroblasts exhibit higher sensitivity to surface tension on the culture substrate and lower tolerance to mechanical stretching in terms of cell morphology and F-actin and α-tubulin protein expression. Furthermore, we found that 17-β-estradiol (E_2_) can improve POP prognosis by inhibiting the mechanical stretching-induced overexpression of F-actin and α-tubulin in healthy fibroblasts and decreased expression of these proteins in POP fibroblasts, thus restraining cell deformation [8]. The ECM is mainly composed of collagens, proteoglycans and glycoproteins. In the present study, we first observed the effects of a 0.1-Hz uniaxial CS stretching load with 10% elongation on the morphological characteristics of extracellular polymers on the cell surface using scanning electron microscopy (SEM). Collagens are the primary structural components of the ECM, with type I collagen (COL I) and type III collagen (COL III) being responsible for mechanical strength and tissue elasticity, respectively [9,10]. Moreover, collagens are the predominant proteins in vaginal connective tissues [11]. Thus, we wanted to further investigate the effects of a 0.1-Hz uniaxial CS load with 10% elongation on COL I and COL III mRNA expression using real-time quantitative polymerase chain reaction (RT-PCR). Small leucine-rich proteoglycans (SLRPs) are components of the ECM that are structurally characterized by a specific protein core with leucine-rich repeat (LRR) motifs substituted with one or more covalently linked glycosaminoglycan (GAG) side chains, and SLRPs are classified into five distinct families based on conservation and homology at the protein and genomic levels, the presence of characteristic N-terminal cysteine-rich clusters with defined spacing, and chromosomal organization [12,13]. SLRPs are capable of binding different cell-surface receptors, cytokines, growth factors, and other ECM components to modulate cellular functions, such as collagen fibrillogenesis and matrix assembly, cell proliferation and differentiation, innate immunity and inflammation, and tumor growth and metastasis [14–17]. Decorin (DCN), biglycan (BGN), fibromodulin (FMO), and lumican (LUM) are the best-characterized members of the SLRP family and have been extensively studied. DCN was found to inhibit downstream oncogenic signaling in several solid tumors by binding and antagonizing various receptor tyrosine kinases [18]; LUM was reported to have anticancer activity by effectively regulating the estrogen receptor-associated functional properties of breast cancer cells, the expression of matrix effectors, and the epithelial-to-mesenchymal transition [19].

As biologically active components of the ECM, SLRPs work in concert to assemble collagen fibrils into a functioning ECM, contribute to the development of connective tissue mechanical properties [20], and protect collagen fibrils from proteolytic cleavage by various collagenases and thus play roles in guiding and stabilizing the formation and maturation of collagen fibrils [21,22]. Altered expression and structural deficiency of SLRPs impact matrix assembly and tissue function. Knockout of both DCN and BGN genes leads to larger and heterogeneous fibril diameters in tendons, and acute ablation results in tendon failure at lower loads, as well as decreased stiffness [23]. Alterations were observed early in LUM-deficient tendons, and a severe phenotype was acquired at maturation in FMD-deficient tendons during fibrillogenesis [24]. Therefore, in the present study, we also examined the effects of a 0.1-Hz uniaxial CS load with 10% elongation on DCN, BGN, FMO, and LUM mRNA expression using RT-PCR. We hypothesized that the mechanical stretching caused by the increase in intra-abdominal pressure and gravity applied to the pelvic floor plays an important role in the occurrence and progression of POP. In turn, the excessive mechanical stretching load may impair the integrity and mechanical properties of the pelvic supportive connective tissues by changing the secretion and organization of the reconstructed ECM, including COL I and COL III and the SLRP members DCN, BGN, FMO, and LUM, eventually resulting in POP. Thus, the purpose of the present study was to investigate the mechanism of POP by observing the effects of a 0.1-Hz uniaxial CS load with 10% elongation on the morphological characteristics of extracellular biopolymers on the cell surface and on the mRNA expression of COL I and COL III as well as the SLRP members DCN, BGN, FMO, and LUM in POP fibroblasts in vitro. Furthermore, we aimed to evaluate the effect of estrogen therapy (ET) in association with the improvement of the prognosis of POP.

## Materials and methods

### Patient selection and tissue collection

This study was approved by the medical ethics committee of Beijing Chaoyang Hospital, Capital Medical University, on January 23, 2013 (project identification code: 13-S-11). From January 24, 2013 to December 26, 2013, a total of 12 participants were recruited. Six women (aged 52–69 years) undergoing pelvic floor construction surgery with advanced POP (stage III-IV by POP quantification) constituted the case group, and six women (aged 49–67 years) undergoing benign gynecologic hysterectomy due to fibroids, dysfunctional bleeding, or ovarian cysts were the controls. In addition to the influence of innate immunity, inflammation, and malignant tumors on SLRP expression as described above [13–19], endometriosis is a disease involving chronic inflammation and fibrosis of the parametrium and uterine ligaments [25]. To avoid the interference of these diseases on the experimental results, patients with endometriosis, gynecologic malignancies, pelvic inflammatory conditions, connective tissue disorders, or emphysema were excluded. All participants provided verbal and written informed consent. After informed consent was obtained, a 1-cm^2^, full-thickness area of the vaginal wall was procured from the anterior wall near the vaginal apex of the POP and control patients during surgery. The connective tissue beneath the anterior vaginal wall approximates the vaginal fornix, which was previously considered to be a representative portion of the endopelvic fascia [26].

### Primary culture of human vaginal fibroblasts

The procedures used to isolate and culture vaginal connective tissue fibroblasts were the same as those described in our previous report [8]. Briefly, the excised vaginal wall samples were immediately placed in 4°C sterile Dulbecco’s phosphate-buffered saline (DPBS, HyClone, South Logan, UT, USA) with 1% penicillin/streptomycin (HyClone, South Logan, UT, USA) and sent to the laboratory within 2 h. Connective tissue blocks were first separated from the vaginal wall samples and minced into 1-mm^3^ pieces and then digested for 24 h with 0.5% collagenase type I (Sigma-Aldrich, St. Louis, MO, USA) in Dulbecco’s modified Eagle’s medium (Gibco, Grand Island, NY, USA) in a 5% CO_2_ humidified incubator at 37°C. Finally, the fine sand-like tissue pieces resulting from collagenase digestion were suspended and centrifuged, and the sediment was reconstituted and cultured in Dulbecco’s modified Eagle’s medium (supplemented with 10% fetal bovine serum and 1% penicillin/streptomycin).

### Immunohistochemical identification of fibroblasts and immunofluorescence analysis of collagens and SLRPs

At the fourth passage, the derived cells were identified using the immunohistochemical streptavidin-peroxidase (SP) method, and the staining results were assessed using an index of staining (IS), as in our previous report [8]. Cells at 50% confluence cultured in chamber slides were fixed with 4% paraformaldehyde, treated with 0.4% Triton X-100, blocked with 3% hydrogen peroxide, and then incubated overnight with mouse anti-human vimentin monoclonal antibody (1:200), mouse anti-human cytokeratin monoclonal antibody (1:200), mouse anti-human α-smooth muscle actin monoclonal antibody (1:50), or DPBS (as the negative control) at 37°C (all antibodies were from Zhongshan Goldbridge Biotechnology, Beijing, China). Subsequently, the cells were incubated with the PV-6000 polymer detection system for immunohistological staining (Zhongshan Goldbridge Biotechnology, Beijing, China), and immunoreactivities were revealed using a 3,3′-diaminobenzidine tetrahydrochloride substrate kit (Zhongshan Goldbridge Biotechnology, Beijing, China). In addition, the cells were counterstained with Meyer’s hematoxylin and finally identified by examining the IS values.

COL I, COL III, BGN, DCN, FMO, and LUM protein expression in the isolated fibroblasts was confirmed by immunofluorescent staining according to the suggested protocol of Wen Y [27]. Specifically, cells at 50% confluence cultured in chamber slides were fixed with 4% paraformaldehyde, treated with 0.4% Triton X-100, and then incubated overnight with different conjugated primary antibodies, including FITC-conjugated rabbit anti-collagen (I or III) polyclonal IgG antibody (1:200), rhodamine-conjugated rabbit anti-DCN (or anti-BGN, anti-FMO, or anti-LUM) polyclonal IgG antibody (1:200), or DPBS (as the negative control) at 37°C (all antibodies were from Bioss, Beijing, China). Finally, nuclei were stained with Hoechst 33342 (Enzo Life Sciences, Farmingdale, NY, USA), and the cells were observed on an Olympus BX51 fluorescence microscope equipped with an Olympus DP72 camera (Olympus Optical Co Ltd, Tokyo, Japan).

### Mechanical stretching and E_2_

Mechanical stretching experiments were performed according to our previous study using a cell-stretching device designed and manufactured to apply stress in vitro [8]. The identified fourth-passage fibroblasts were seeded on a gelatin-coated polydimethylsiloxane membrane with a utilized area of 40 × 20 mm^2^ (length × width) and a thickness of 3 mm at a density of 2 × 10^3^ cells/cm^2^ to assess cell morphology and a density of 2 × 10^4^ cells/cm^2^ to assay mRNA expression. After being cultured for 24 h, a 0.1-Hz uniaxial CS with 10% elongation and a 12-h stretching duration was applied to the vaginal fibroblasts every day with or without a concomitant dose of 10^−8^ M E_2_ (Sigma-Aldrich Co, St. Louis, MO, USA) for 72 h. Non-stretched vaginal fibroblasts cultured on gelatin-coated polydimethylsiloxane membranes were used as controls. The viable cells in each group were collected to perform SEM analysis and mRNA detection.

### Imaging of extracellular polymers via SEM

SEM analysis was performed to observe the CS-induced changes in cell shape and extracellular polymer structure. For this purpose, cells were washed with DPBS (pH 7.4) and fixed with 2% glutaraldehyde in DPBS (pH 7.4) for 4 h at 4°C. The specimens were dehydrated using an ascending ethanol gradient (50%, 70%, 80%, 90% and 100%), after which the ethanol was replaced with tertbutyl alcohol. After dehydration, the specimens were critical-point dried with CO_2_. Finally, the specimens were sputter-coated with gold in an ion coater for 2 min at an applied current of 50 mA (Eiko IB-3, Eiko Engineering Ltd, Tokyo, Japan) and examined via SEM (S-570, Hitachi, Japan).

### RT-PCR

RNA was extracted from the experimental cells using an RNeasy Mini Kit (Qiagen, Hilden, Germany) according to the manufacturer’s standard protocol. The RNA concentration was controlled to an OD260/OD280 ratio of >1.8 using a NanoDrop 2000 spectrophotometer (Thermo Scientific, Wilmington, DE, USA).

Reverse transcription was performed using a QuantiTect Reverse Transcription Kit (Qiagen, Hilden, Germany) following the supplier’s recommendations. Briefly, a 20-μL reaction volume, including 1 μg of total RNA, 1 μL of oligo (dT)_15_ (10 μM, TIANGEN, Beijing, China), and 10 μL of 2× RT-PCR Buffer (Qiagen, Hilden, Germany), was heated to 65°C for 5 min, quickly chilled, and incubated at 42°C for 1 h to allow the reaction, after which the enzyme was inactivated by heating at 85°C for 1 min. The oligonucleotide primer sequences for COL I and COL III were described previously [28], as were those for FMO, DCN, and BGN [27]. We designed the primer sequences for LUM. The housekeeping gene 18S was used as a reference. All the primers were validated using NCBI Primer-BLAST and synthesized by TaKaRa (TaKaRa Biotechnology, Dalian, China) and are listed in Table 1.

**Table 1:** The primers for collagens, SLRPs, and the housekeeping gene 18S.

Gene	Oligo	Primer sequence	Product size (bp)
**COL I**	Sense	5’-TCCCCAGCCACAAAGAGTCTACA-3’	156
	Anti-sense	5’-GTGATTGGGTGGGATGTCTTCGTC-3’	
**COL III**	Sense	5’-CTGCCATCCTGAACTCAAGAGTGG-3’	447
	Anti-sense	5’-CCATCCTCCAGAACTGTGTAGG-3’	
**DCN**	Sense	5’-CTGATGACCGCGACTT-3’	132
	Anti-sense	5’-GAGTTGTGTCAGGGGGAAGA-3’	
**BGN**	Sense	5’-TGTTCCCTCCATCTCTCCGAACCTG-3’	141
	Anti-sense	5’-GACCGCTGTCCCTGGGGTTTTG-3’	
**FMO**	Sense	5’-GGGGCAAGGACTGTTGGAGGAG-3’	146
	Anti-sense	5’-CCAGGTCTGGAGCCAAGAACGTAGT-3’	
**LUM**	Sense	5’-ATCACTCAGAATCTGGCAGC-3’	267
	Anti-sense	5’-CAGTTACATTCTGGTGCACAG-3’	
**18S**	Sense	5’-CAGCCACCCGAGATTGAGCA-3’	252
	Anti-sense	5’-TAGTAGCGACGGGCGGTGTG-3’	

RT-PCR was performed using a QuantiTect SYBR Green RT-PCR Kit (Qiagen, Hilden, Germany) and an Applied Biosystems 7500 Real-Time PCR System (Applied Biosystems, Foster City, CA, USA). Each RT-PCR reaction was performed in a 20-μL total volume containing 10 μL of 2× QuantiTect SYBR Green RT-PCR master mix, 8 μL of 1:4 diluted cDNA template, and 0.5 μL of each of the forward and reverse target-specific primers (10 μmol/L), which were designed to amplify a part of each gene. Amplification was performed as follows: 50°C for 2 min; 95°C for 15 min; and 45 cycles of 94°C for 15 sec and 55°C for 30 sec, followed by 72°C for 40 sec. After PCR, a melting-curve analysis was performed to demonstrate the specificity of the PCR product through the presence of a single peak. A control reaction containing all of the reaction components except for the template was included in all experiments. RT-PCR assays were validated as described previously [29].

### Statistical analysis

Data analysis was carried out using ABI 7500 SDS System software (version 1.4) (Applied Biosystems, Foster City, CA, USA). COL I, COL III, DCN, BGN, FMO, and LUM mRNA expression levels were normalized to that of the gene 18S based on ΔCt = Ct for the gene of interest—Ct for the housekeeping gene. The data were analyzed using the 2^-ΔΔCt^ method and are presented as the mean ± standard error (SE); the baseline values were considered to be 1. Statistical analyses were conducted using SPSS for Windows (SPSS, Chicago, IL, USA). Comparisons of multiple groups were performed using one-way analysis of variance, and differences between two groups were determined using Student’s t-test. The results were considered significant at *P* < 0.05. To guarantee the accuracy of the results, each experiment was performed in triplicate, each independent experiment involved three rounds of RT-PCR detection, and the researcher who collected and processed the original data could not identify individual participants during or after data collection.

## Results

### Morphological characteristics of vaginal fibroblasts

**Fig 1:**
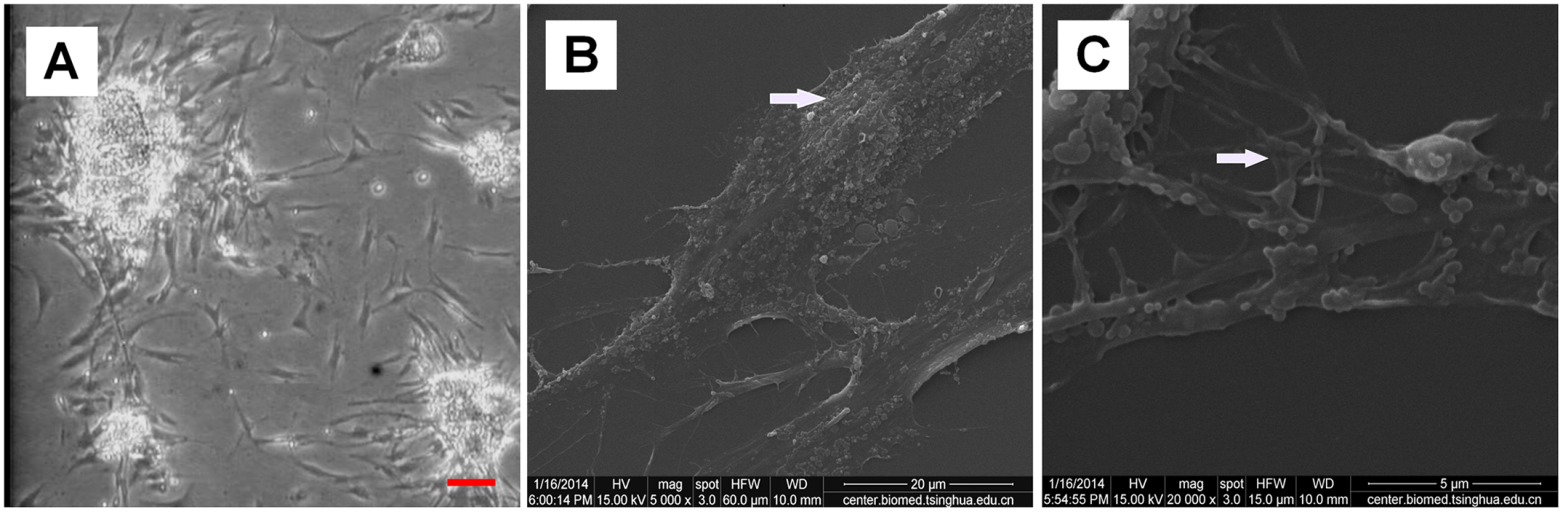
Morphological characteristics of vaginal fibroblasts under static conditions. (A) Primary cultured normal fibroblasts after 72 h at passage 0 (bar = 100 μm). (B) Slightly raised nuclear area of normal fibroblasts at passage 4 (bar = 20 μm). (C) Filopodial connections between adjacent cells of normal fibroblasts at passage 4 (bar = 5 μm). Images were acquired on an inverted microscope (A) and SEM (B) (C).

Fibroblasts isolated from the vaginal wall connective tissues of women with POP and unaffected women all exhibited a stellate, bipolar, or spindle-shaped morphology when observed on an inverted microscope (Fig 1A). When observed using SEM, the fibroblasts exhibited an obvious long fusiform shape with a slightly raised nuclear area (Fig 1B) and coarse protrusions or slender filopodia that formed connections between adjacent cells (Fig 1C).

### Immunohistochemical and immunofluorescence assays in vaginal fibroblasts

SP staining of all fibroblasts isolated from the vaginal wall connective tissues of women with and without POP showed strong cytoplasmic expression of vimentin, with an IS of 12 (+++); negative cytoplasmic expression of cytokeratin, with an IS of 0 (−); negative cytoplasmic expression of α-smooth muscle actin, with an IS of 0 to 1 (−); and negative cytoplasmic expression of the negative control, with an IS of 0 (−). These findings revealed that the isolated fibroblasts in this study were of connective tissue origin.

**Fig 2:**
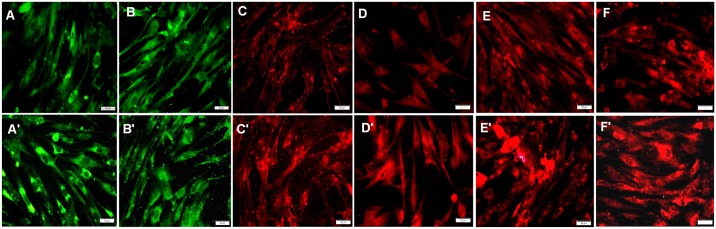
Immunofluorescence analysis indicating the expression of collagens and SLRPs in vaginal fibroblasts. (A) Green staining for COL I in normal fibroblasts. (A') Green staining for COL I in POP fibroblasts. (B) Green staining for COL III in normal fibroblasts. (B') Green staining for COL III in POP fibroblasts. (C) Red staining for DCN in normal fibroblasts. (C') Red staining for DCN in POP fibroblasts. (D) Red staining for BGN in normal fibroblasts. (D') Red staining for BGN in POP fibroblasts. (E) Red staining for FMO in normal fibroblasts. (E') Red staining for FMO in POP fibroblasts. (F) Red staining for LUM in normal fibroblasts. (F') Red staining for LUM in POP fibroblasts. Bar = 50 μm.

Immunofluorescent staining for COL I, COL III, DCN, BGN, FMO, and LUM in vaginal fibroblasts from the unaffected and POP groups was also performed. Fibroblasts from both groups showed green immunofluorescent staining patterns for COL I (Fig 2A and 2A') and COL III (Fig 2B and 2B') and red immunofluorescent staining patterns for DCN (Fig 2C and 2C'), BGN (Fig 2D and 2D'), FMO (Fig 2E and 2E'), and LUM (Fig 2F and 2F'), confirming the presence and distribution of these proteins in fibroblasts from the two groups. The identified fibroblasts were used at the fifth passage in the following experiments and were divided into 8 groups: a normal group (N) and a POP group (P) without CS or E_2_ [(N-E_2_-CS) and (P-E_2_-CS)], groups C and P without E_2_ but with CS [(N-E_2_+CS) and (P-E_2_+CS)], groups C and P with E_2_ but without CS [(N+E_2_-CS) and (P+E_2_-CS)], and groups C and P with both CS and E_2_ [(N+E_2_+CS) and (P+E_2_+CS)].

### Effects of CS on morphological characteristics of fibroblasts and extracellular polymers

**Fig 3:**
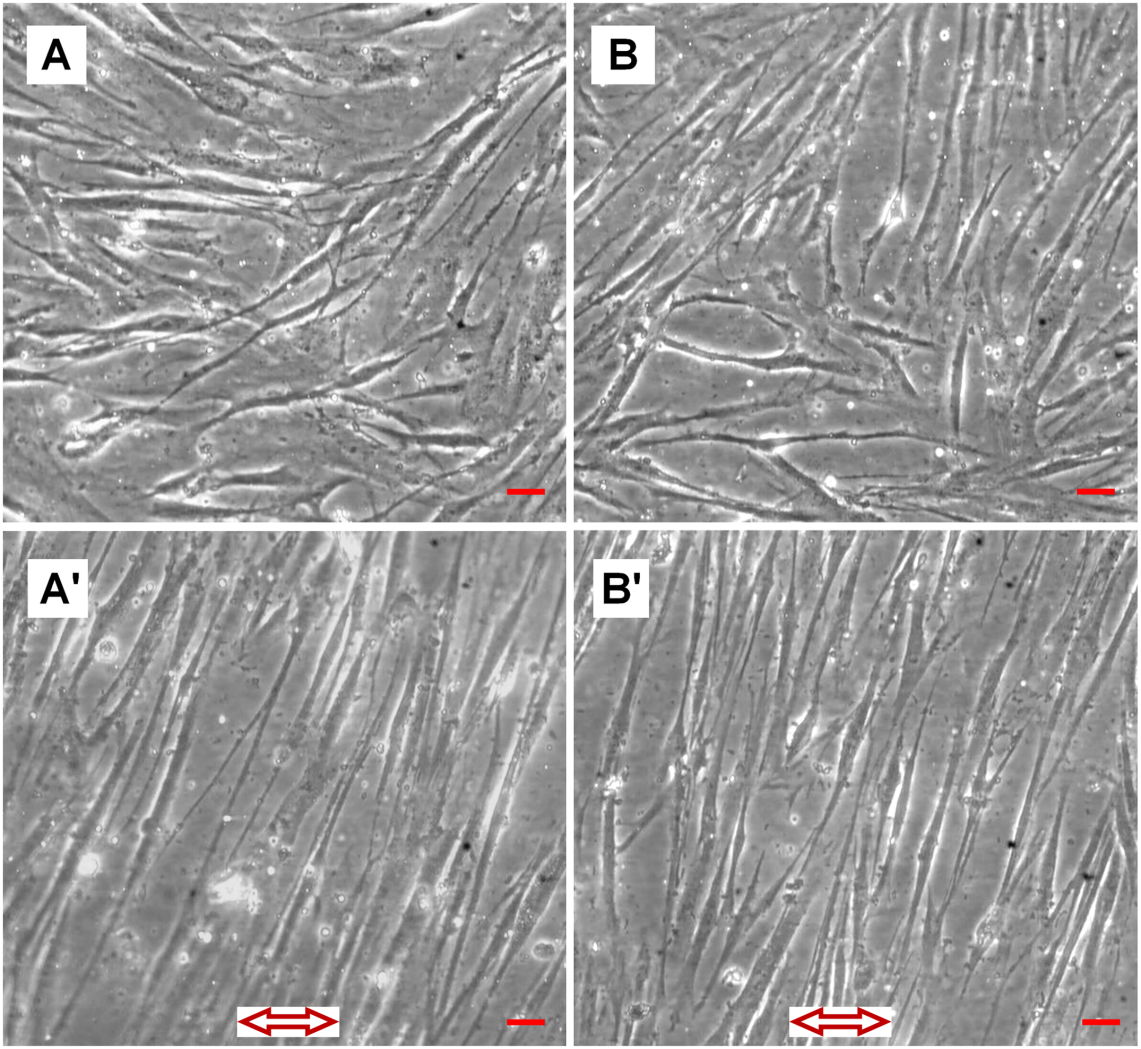
Morphological characteristics of vaginal fibroblasts and effects of CS, as observed by light microscopy. (A) Static-cultured normal fibroblasts with a random swirling distribution. (B) Static-cultured POP fibroblasts with a random swirling distribution. (A') Stretched normal fibroblasts aligned perpendicular to the force. (B') Stretched POP fibroblasts aligned perpendicular to the force. Bar = 100 μm. The arrow indicates the stretching direction.

**Fig 4:**
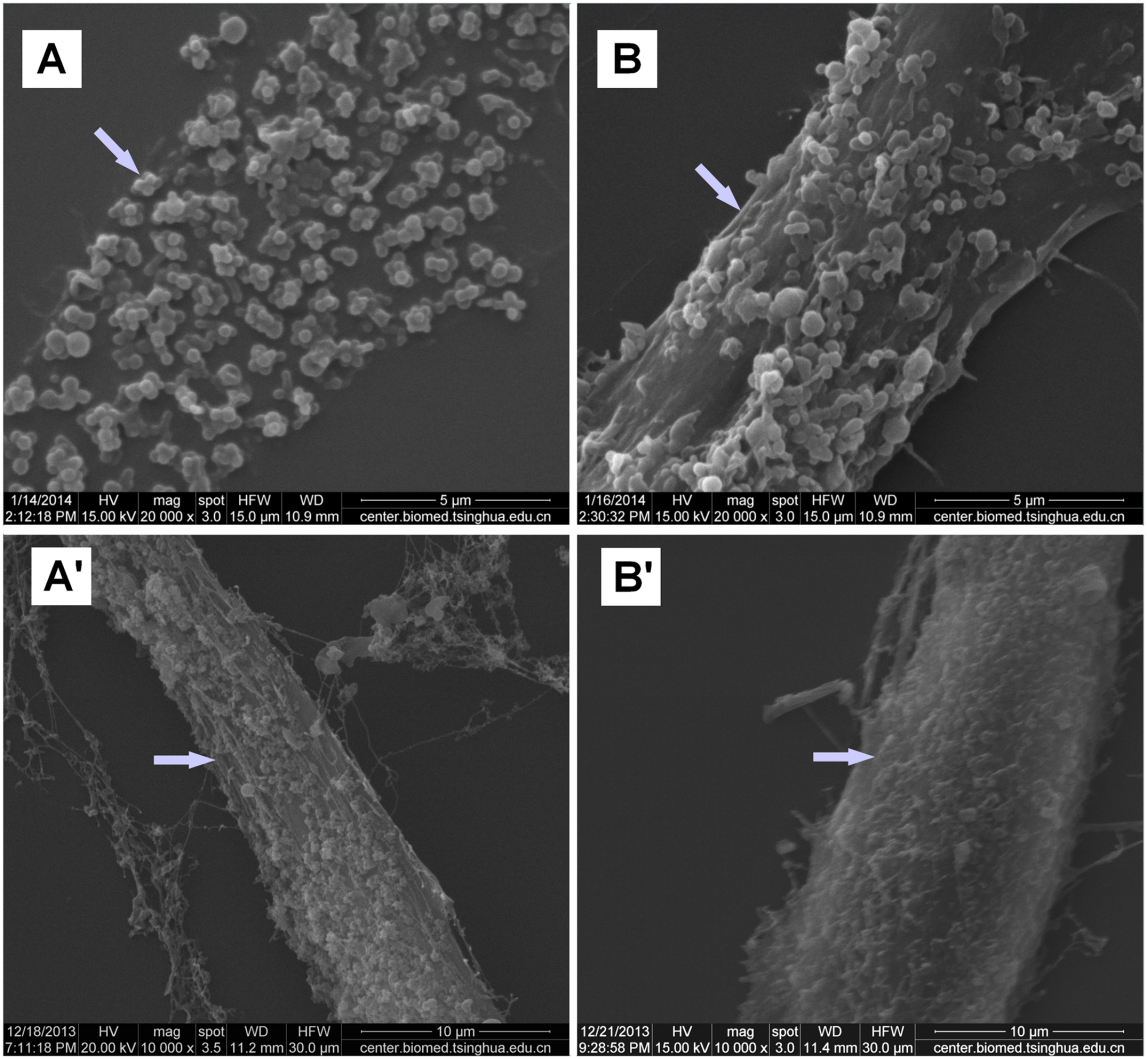
Effects of CS on the characteristics of extracellular polymers, as observed via SEM. (A) Plum flower-like distribution of polymers on normal fibroblast surfaces. (B) Stretched strip-like distribution of polymers on POP fibroblast surfaces. (A') Stretched strip-like distribution of polymers on stretched normal fibroblast surfaces. (B') Broken network-like distribution of polymers on stretched POP fibroblast surfaces.

When viewed on a light microscope, static-cultured fibroblasts presented a randomly swirling distribution (Fig 3A and 3B), whereas stretching-cultured fibroblasts grew perpendicular to the force (Fig 3A' and 3B'), with no significant differences in cell surface morphology observed between normal and POP fibroblasts. When observed via SEM, some polymers secreted by the fibroblasts were distributed on the cell surface. Under static-culture conditions, the polymers on the normal fibroblast surfaces were randomly distributed in clusters of plum flower-like patterns (Fig 4A), whereas on the POP fibroblast surfaces, the polymers resembled stretched strips, with stretching along the long axis of the cells (Fig 4B). When the cells were stretched, the polymers on the normal fibroblast surfaces also resembled stretched strips, again with stretching along the long axis of the cells (Fig 4A'), whereas those on the POP fibroblast surfaces resembled a broken network (Fig 4B').

### Effects of CS and E_2_ on collagen and SLRP mRNA expression in vaginal fibroblasts

**Fig 5:**
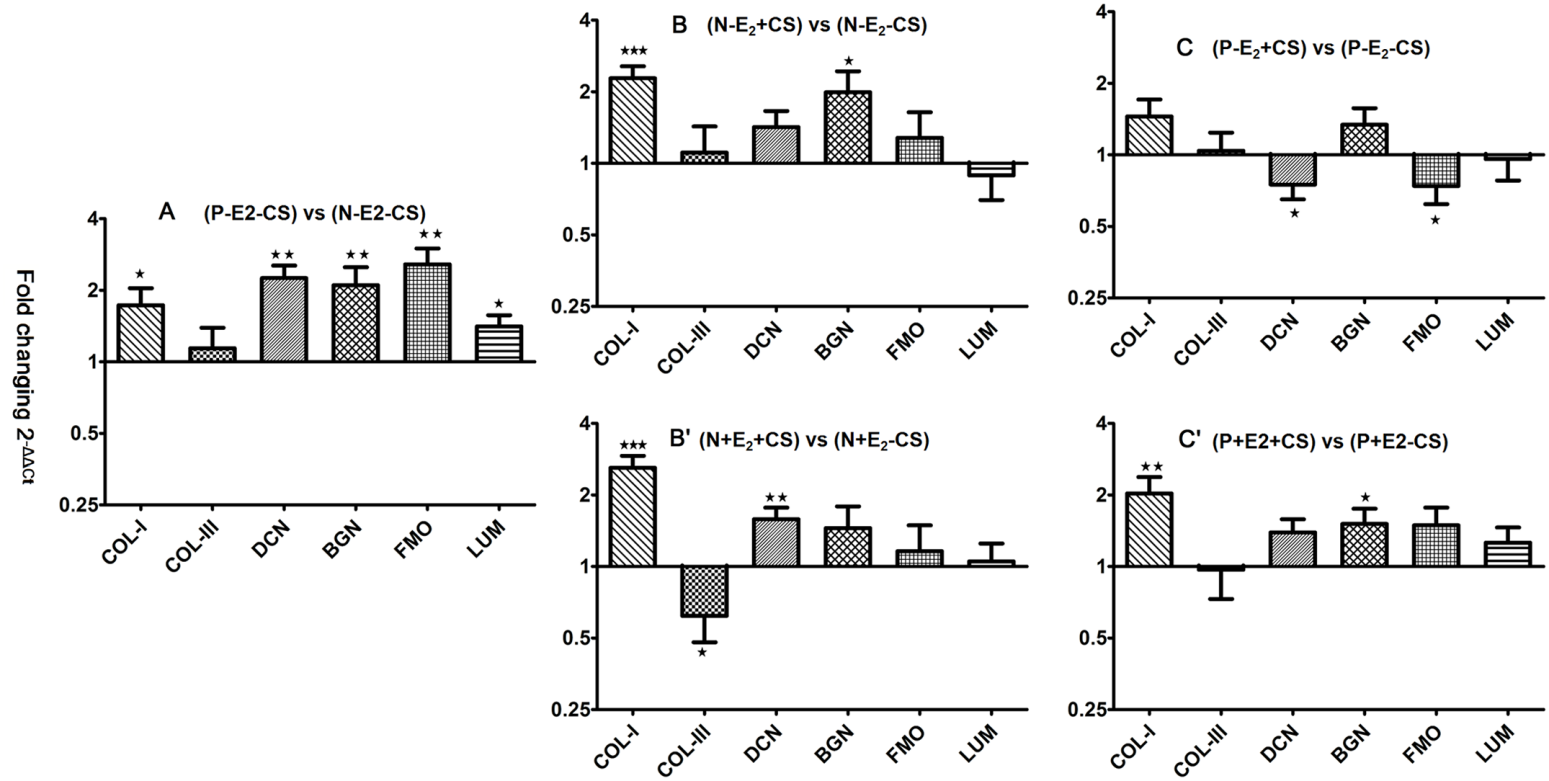
Effects of CS and E_2_ on collagen and SLRP mRNA expression in vaginal fibroblasts. The bars and error bars indicate the mean and SE, respectively. Data represent the mean ± SE of triplicate trials. * *P* < 0.05; **P < 0.01, ***P< 0.001.

Compared with (N-E_2_-CS) group, (P-E_2_-CS) group exhibited significantly higher COL I mRNA expression (2^-ΔΔCt^ = 1.73 ± 0.31, *P* = 0.030). Similar differences in mRNA expression were noted for DCN, BGN, FMO, and LUM between these two groups (2^-ΔΔCt^ = 2.25 ± 0.29, *P* = 0.001; 2^-ΔΔCt^ = 2.10 ± 0.40, *P* = 0.014; 2^-ΔΔCt^ = 2.57 ± 0.43, *P* = 0.002; 2^-ΔΔCt^ = 1.41 ± 0.16, *P* = 0.022), whereas no significant difference was observed in COL III mRNA expression between the two groups (2^-ΔΔCt^ = 1.14 ± 0.25, *P* = 0.594) (Fig 5A).

Following the application of stretching, normal fibroblasts exhibited a significant increase in COL I mRNA expression, with a significant difference specifically being found between the (N-E_2_+CS) and (N-E_2_-CS) group (2^-ΔΔCt^ = 2.28 ± 0.28, *P* < 0.001). A similar difference in BGN mRNA expression was observed between these two groups (2^-ΔΔCt^ = 1.99 ± 0.45, *P* = 0.041), whereas no significant difference was observed in COL III, DCN, FMO, or LUM mRNA expression in the (N-E_2_+CS) group compared with the (N-E_2_-CS) group (2^-ΔΔCt^ = 1.10 ± 0.32, *P* = 0.746; 2^-ΔΔCt^ = 1.42 ± 0.24, *P* = 0.096; 2^-ΔΔCt^ = 1.28 ± 0.36, *P* = 0.439; 2^-ΔΔCt^ = 0.89 ± 0.19, *P* = 0.552) (Fig 5B). However, POP fibroblasts subjected to stretching force exhibited no significant increase in COL I mRNA expression, with the data specifically revealing no significant difference between the (P-E_2_+CS) and (P-E_2_-CS) groups (2^-ΔΔCt^ = 1.45 ± 0.26, *P* = 0.105). In contrast, significant decreases in DCN and FMO mRNA expression were found in the (P-E_2_+CS) group compared with the (P-E_2_-CS) group (2^-ΔΔCt^ = 0.75 ± 0.10, *P* = 0.019; 2^-ΔΔCt^ = 0.74 ± 0.12, *P* = 0.037), whereas no significant difference was observed in COL III, BGN, or LUM mRNA expression between the (P-E_2_+CS) and (P-E_2_-CS) groups (2^-ΔΔCt^ = 1.04 ± 0.20, *P* = 0.859; 2^-ΔΔCt^ = 1.34 ± 0.23, *P* = 0.152; 2^-ΔΔCt^ = 0.96 ± 0.18, *P* = 0.816) (Fig 5C).

When cultured in the presence of concomitant E_2_ treatment and stretching, the normal fibroblasts still exhibited significantly increased COL I mRNA expression, with a significant difference specifically being found between the (N+E_2_+CS) and (N+E_2_-CS) groups (2^-ΔΔCt^ = 2.60 ± 0.32, *P*< 0.001), whereas COL III mRNA expression was significantly lower in the (N+E_2_+CS) group than in the (N+E_2_-CS) group (2^-ΔΔCt^ = 0.62 ± 0.14, *P* = 0.017). In addition, DCN mRNA expression was significantly increased in the (N+E_2_+CS) group compared with the (N+E_2_-CS) group (2^-ΔΔCt^ = 1.58 ± 0.19, *P* = 0.009), whereas no significant difference was observed in BGN, FMO or LUM mRNA expression between the (N+E_2_+CS) and (N+E_2_-CS) groups (2^-ΔΔCt^ = 1.45 ± 0.34, *P* = 0.201; 2^-ΔΔCt^ = 1.16 ± 0.33, *P* = 0.637; 2^-ΔΔCt^ = 1.05 ± 0.20, *P* = 0.789) (Fig 5B'). With respect to the effects of concomitant E_2_ exposure and stretching on POP fibroblasts, both COL I and BGN showed significantly higher mRNA expression in POP fibroblasts in the presence of E_2_ and stretching, with significant differences observed between the (P+E_2_+CS) and (P+E_2_-CS) groups (2^-ΔΔCt^ = 2.03 ± 0.35, *P* = 0.009; 2^-ΔΔCt^ = 1.51 ± 0.24, *P* = 0.047). Moreover, the application of E_2_ abrogated the decrease in the DCN and FMO mRNA expression induced by stretching, with the data indicating a non-significant difference between the (P+E_2_+CS) and (P+E_2_-CS) groups (2^-ΔΔCt^ = 1.39 ± 0.19, *P* = 0.054; 2^-ΔΔCt^ = 1.49 ± 0.28, *P* = 0.097). In contrast, COL III, FMO and LUM mRNA expression was not significantly different between the (P+E_2_+CS) group and the (P+E_2_-CS) group (2^-ΔΔCt^ = 0.97 ± 0.24, *P* = 0.915; 2^-ΔΔCt^ = 1.49 ± 0.28, *P* = 0.097; 2^-ΔΔCt^ = 1.26 ± 0.20, *P* = 0.225) and was similar to that observed in normal fibroblasts under stretching without E_2_ application (Fig 5C').

## Discussion

In the present study, both POP and healthy fibroblasts grew randomly under static-cultured conditions and were arranged perpendicular to the force when under mechanical stretching when viewed on a light microscope, as reported previously [8,30]. To date, no significant difference in the cell surface morphological characteristics between these two groups has been observed by light microscopy after mechanical stretching. Using SEM, however, we observed that certain polymers have a distribution pattern that resembled clusters of plum flowers in healthy fibroblasts and stretched strips in POP fibroblasts under static-cultured conditions, and these biopolymers became arranged perpendicular to the force, clearly resembling stretched strips in healthy fibroblasts and a broken network in POP fibroblasts under the same CS conditions. This result suggests that extracellular polymers secreted on the cell surface by fibroblasts possess viscoelastic properties and that of POP fibroblasts have higher mechanosensitivity to tension on the surface of the culture substrate and lower tolerance to mechanical stretching. The identity of these viscoelastic polymers still needs to be determined; undoubtedly, further investigation of ECM molecules that are secreted by vaginal fibroblasts needs to be conducted. The ECM is a highly organized network composed of various polymers, which contribute viscoelastic properties to connective tissues. Collagen molecules, which form a triple-helix structure comprising three α chains, are the major components of extracellular viscoelastic polymers [31]. The rupture/formation of H-bonds between the three collagen chains is the primary mechanism of the viscoelastic behavior [32]. Moreover, in the present study, we investigated the effects of mechanical stretching on the mRNA expression of the structural ECM proteins COL I and COL III in vaginal fibroblasts. Due to their ability to form molecular bridges between neighboring collagen fibrils and to absorb a large amount of water, SLRPs may also endow ECM with viscoelasticity. We therefore also investigated the mRNA expression of the SLRP family members DCN, BGN, FMO, and LUM in vaginal fibroblasts. Hyaluronan, another proteoglycan with a high affinity for water, is also considered an important viscoelastic component of the ECM [33], and its metabolism and ratio to collagens needs to be examined in further studies.

As mechanosensitive cells, fibroblasts can sense strain applied to the ECM and convey this information to the nucleus via the cytoskeleton, ultimately mounting an adaptive response by increasing or decreasing the production of ECM proteins, including collagens and proteoglycans, to maintain the morphology and function of connective tissues [34–36]. Bildircin D et al [37] reported significantly higher COL I content in POP uterine ligament fibroblasts than in the control group, whereas the COL III content did not significantly differ. Another study reported that uniaxial CS can increase COL I or COL III mRNA expression in anterior cruciate ligament fibroblasts [38], while another study reported that uniaxial CS with a frequency of 0.5 Hz and a magnitude of 4% or 8% applied for 4 h followed by resting for 20 h was able to increase COL I gene and protein expression but only slightly increased COL III gene expression levels in human patellar tendon fibroblasts [39]. In the current study, we detected higher COL I mRNA expression in POP fibroblasts under static-culture conditions and confirmed that a uniaxial CS load with a frequency of 0.1 Hz and a magnitude of 10% can increase COL I mRNA expression in healthy fibroblasts but not in POP fibroblasts. This finding also corresponds to the higher sensitivity of POP fibroblasts to mechanical stimuli on the surface of the culture substrate and their lower tolerance to mechanical stretching in terms of COL I transcription. We also did not observe any significant difference in COL III gene expression between POP and healthy fibroblasts under static-culture conditions in the present study, and mechanical stretching did not affect COL III gene expression in either group. These findings for COL III may be due to the fact that COL III is relatively enriched in loose connective tissue, whereas the supportive connective tissues of the pelvic floor comprises dense connective tissue that mainly expresses COL I. As COL I plays a major role in maintaining tissue strength while COL III mainly confers elasticity to connective tissues, the changes in COL I and COL III expression observed in the present study may indicate that tensile strength, and not elasticity, is damaged in the supportive connective tissues in POP.

Four important SLRP members with a collagen-regulating function, namely, DCN, BGN, FMO, and LUM, were also investigated in this study, and for the first time, we showed the different effects of a mechanical stretching load on these proteins between POP and healthy fibroblasts. Both DCN and BGN belong to the class I subfamily of SLRPs that contain chondroitin/dermatan sulfates, with DCN carrying only one and BGN most often carrying two GAG chains, whereas both FMO and LUM are class II SLRP members with one keratan sulfate side chain [12,40]. Structural similarities indicate that DCN and BGN competitively bind to adjacent sites on COL I [41], whereas FMO and LUM competitively bind to the same regions of COL I. DCN binds to collagen primarily via LRRs 4–5, composed of approximately 40 amino acid residues, and the conformation of the collagen-binding LRRs 4–5 is different in DCN and in the BGN/DCN chimera, leading to a lower collagen affinity for BGN [42]. The sequence homology between LUM and FMO LRRs 5–7 results in FMO and LUM competing for the same binding site on COL I, and binding competition experiments and Scatchard plot analysis indicate that FMO binds to COL I with a higher affinity than LUM. The collagen-binding site in the FMO LRRs 5–7 inhibits LUM binding to COL I [43,44]. However, the binding sites for FMO or LUM on COL I are separate from the DCN binding sites on collagen, and FMO and LUM do not affect the binding of DCN to collagen, and DCN does not inhibit the binding of FMO or LUM [45,46]. The GAG chains of fibril-bound DCN form antiparallel duplexes with each other to regulate the distance between adjacent collagen fibrils, while the analogous binding of BGN and collagen adds a further dimension by forming bridges to two separate collagen fibrils; in this context, DCN is the key regulatory molecule [47,48]. DCN-deficient tendons have been reported to develop abnormal, irregularly contoured fibrils, and their strength or stiffness is significantly reduced [49]. Meanwhile, the expression and deposition of BGN can be up-regulated to compensate for the loss of the regulatory role of DCN to maintain relatively normal fibrillogenesis, whereas the corneas of compound DCN/BGN-null mice showed severe disruption of fibril structure and organization [50]. FMO and LUM also interact with fibrillar collagens and play a role in the assembly of collagens into higher-order fibrils in connective tissues [51]. An absence of FMO has been reported to increase the number of immature and thin collagen fibrils and result in reduced tendon stiffness, whereas increased LUM deposition compensates for FMO deficiency to prevent further loss of tendon strength, with the collagen structure and tendon strength of LUM-FMO double-deficient mice being badly affected as a result [52]. LUM and DCN have synergistic effects in modulating collagen fibrillogenesis; LUM accelerates initial fibril formation while DCN retards initial fibril formation, and both also enhance collagen fibril stability [45]. Our data revealed that DCN, BGN, FMO, and LUM mRNA expression were higher in POP fibroblasts than in healthy fibroblasts under static-culture conditions. These results suggest higher mechanosensitivity to surface tension, resulting in transcription of these SLRPs in POP fibroblasts and a role for these proteins in the structure and function of COL I following increased expression. Following application of the CS load in this study, DCN and FMO mRNA expression did not change significantly in healthy fibroblasts, but BGN mRNA expression increased and was accompanied by up-regulation of COL I. In contrast, DCN and FMO mRNA expression was significantly down-regulated in POP fibroblasts, and this down-regulation could not be compensated for by an increase in BGN or LUM. These findings suggest that POP fibroblasts also possess lower tolerance to mechanical stretching in terms of DCN and FMO transcription, as well as defects in the compensatory function of BGN for DCN and LUM for FMO. This compensatory defect may affect the structure and function of COL I in response to mechanical stretching, resulting in a reduction in the strength and stiffness of the pelvic supportive connective tissues and the occurrence of POP. In addition, because LUM influences only the initial assembly of intermediates and the entry into fibril growth, while FMO also facilitates the progression through growth steps leading to mature fibrils [24], no-significant changes in LUM following application of the CS load in this study may indicate that mechanical stretching affects only the mature step of pelvic supportive connective tissue reconstruction.

ET has long been used to improve the symptoms of POP, but the precise effect of estrogen on pelvic-floor structure and its role in the prevention and treatment of POP remain controversial [53,54]. Our previous study suggested that E_2_ improves changes in the cytoskeleton and cell morphology in mechanically stretched healthy and POP fibroblasts to maintain the mechanical properties of both the fibroblasts and the supportive connective tissues of the pelvic floor to further prevent POP occurrence and progression [8]. Therefore, we also explored the actions of E_2_ in the current study. Our data revealed that when E_2_ and CS were concomitantly administered, healthy fibroblasts maintained significantly increased COL I and DCN mRNA expression, and significantly lower COL III mRNA expression was found. These results suggest that when a stretching load is applied to healthy fibroblasts, the use of ET can maintain the integrity of the biomechanical properties of the pelvic supportive connective tissues by up-regulating COL I and by regulating the structure and function of COL I following its increased expression, mainly by up-regulating DCN, to prevent the occurrence of POP. Here, the down-regulation of COL III mRNA expression may indicate that the higher strength and lower elasticity of healthy connective tissues may make it more difficult to stretch these tissues to the point that POP occurs. For POP fibroblasts, our data showed that when E_2_ was administered concomitantly with CS, COL I mRNA expression was significantly increased, accompanied by the up-regulation of BGN mRNA expression, and that DCN and FMO mRNA expression levels were no longer decreased. These findings suggest that once POP has occurred, ET can support the structure and function of COL I by inhibiting the down-regulation of DCN and FMO mRNA expression induced by mechanical stretching and recovering the compensatory function of BGN to improve the mechanical properties of the pelvic supportive connective tissues to inhibit POP progression. LUM expression levels were reported to be significantly decreased in the skin of estrogen receptor-α knockout mice and significantly increased in estrogen receptor-β knockout mice [55]. In the present study, E_2_ did not significantly increase or decrease LUM mRNA expression in healthy or POP fibroblasts when the CS load was applied. This result may be explained by the fact that estrogen receptor-α and estrogen receptor-β are expressed in the vaginal wall of postmenopausal women [56] and that local vaginal ET will increase estrogen receptor-α expression, while estrogen receptor-β expression will remain largely unchanged [57].

The present study further reveals the higher sensitivity and lower tolerance to mechanical stretching in the occurrence and progression of POP through changes in the transcription level of COL I and certain SLRPs, and further confirms previous evidence supporting the use of ET to prevent and inhibit POP in postmenopausal women.

## Conclusions

In conclusion, the morphological distribution of the extracellular polymers on the surface of POP fibroblasts revealed higher sensitivity to tension stimuli and lower tolerance to mechanical stretching. Furthermore, the higher sensitivity to tension stimuli was found to be reflected by the transcription of COL I, and the transcription of DCN, BGN, FMO, and LUM was found to be up-regulated as COL I transcription increased. Meanwhile, POP fibroblasts were found to possess lower tolerance to mechanical stretching in terms of COL I transcription and defects in the compensatory function of BGN for DCN and LUM for FMO, thereby affecting the structure and function of COL I in response to mechanical stretching and ultimately resulting in POP. In healthy women, ET can preserve the integrity of the pelvic supportive connective tissues by up-regulating COL I and DCN transcription and maintaining the normal compensatory function of BGN to preserve the structure and function of COL I following its increased expression to prevent the occurrence of POP. Once POP has occurred, ET can also strengthen the pelvic supportive connective tissues by up-regulating COL I transcription and can support the structure and function of COL I by inhibiting the down-regulation of DCN and FMO transcription induced by mechanical stretching and by recovering the compensatory function of BGN to inhibit the progression of POP. Generally, the higher sensitivity and lower tolerance to stretching possessed by fibroblasts and the metabolism of COL I and certain SLRPs, as well as other ECM components, likely cause the occurrence and progression of POP, and the use of ET will improve prognosis. In future studies, the effects of different CS amplitudes/frequencies on collagen and SLRP expression in fibroblasts from pelvic supportive connective tissues need to be examined, and effective ET concentrations need to be further explored. Furthermore, whether supplementation with certain SLRPs can inhibit the occurrence and development of POP should be investigated.

## References

[1] Hendrix SL, Clark A, Nygaard I, Aragaki A, Barnabei V, McTiernan A. Pelvic organ prolapse in the Women’s Health Initiative: gravity and gravidity. Am J Obstet Gynecol. 2002(6);186:1160–6. 12066091 10.1067/mob.2002.123819

[2] Jelovsek JE, Maher C, Barber MD. Pelvic organ prolapse. Lancet. 2007; 369 (9566):1027–38. doi: 10.1016/S0140-6736(07)60462-0 17382829 10.1016/S0140-6736(07)60462-0

[3] Kim C, Jeon MJ, Chung DJ, Kim SK, Kim JW, Bai SW. Risk factors for pelvic organ prolapse. Int J Gynaecol Obstet. 2007;98(3):248–51. doi: 10.1016/j.ijgo.2007.02.019 17408669 10.1016/j.ijgo.2007.02.019

[4] Word RA, Pathi S, Schaffer JI. Pathophysiology of pelvic organ prolapse. Obstet. Gynecol Clin N Am. 2009;36(3):521–39. doi: 10.1016/j.ogc.2009.09.001 19932414 10.1016/j.ogc.2009.09.001

[5] DeLancey JO. Anatomy and biomechanics of genital prolapse. Clin Obstet Gynecol. 1993;36(4):897–909. 8293591 10.1097/00003081-199312000-00015

[6] Martins P, Lopes Silva-Filho A, Rodrigues Maciel da Fonseca AM, Santos A, Santos L, Mascarenhas T, et al Biomechanical properties of vaginal tissue in women with pelvic organ prolapse. Gynecol Obstet Invest. 2013;75(2):85–92. doi: 10.1159/000343230 23295833 10.1159/000343230

[7] O'Dell KK, Morse AN, Crawford SL, Howard A. Vaginal pressure during lifting, floor exercises, jogging, and use of hydraulic exercise machines. Int Urogynecol J Pelvic Floor Dysfunct. 2007;18(12):1481–9. doi: 10.1007/s00192-007-0387-8 17982711 10.1007/s00192-007-0387-8

[8] Wang S, Zhang Z, Lü D, Xu Q. Effects of mechanical stretching on the morphology and cytoskeleton of vaginal fibroblasts from women with pelvic organ prolapse. Int J Mol Sci. 2015;16(5):9406–19. doi: 10.3390/ijms16059406 25923074 10.3390/ijms16059406 PMC4463595

[9] Gelse K, Poschl E, Aigner T. Collagens–structure, function, and biosynthesis. Adv Drug Deliv Rev. 2003;55(12):1531–46. 14623400 10.1016/j.addr.2003.08.002

[10] Phillips CH, Anthony F, Benyon C, Monga AK. Collagen metabolism in the uterosacral ligaments and vaginal skin of women with uterine prolapse. BJOG. 2006;113(1):39–46. doi: 10.1111/j.1471-0528.2005.00773.x 16398770 10.1111/j.1471-0528.2005.00773.x

[11] Steven DA, Andrew F, Zegbeh J, Pamela AM. Tissue mechanics, animal models, and pelvic organ prolapse: A review. Eur J Obstet Gynecol Reprod Biol. 2009;144(Suppl 1):S146–S158. doi: 10.1016/j.ejogrb.2009.02.022 19285776 10.1016/j.ejogrb.2009.02.022

[12] McEwan PA, Scott PG, Bishop PN, Bella J. Structural correlations in the family of small leucine-rich repeat proteins and proteoglycans. J Struct Biol. 2006;155(2):294–305. doi: 10.1016/j.jsb.2006.01.016 16884925 10.1016/j.jsb.2006.01.016

[13] Schaefer L, Iozzo RV. Biological Functions of the Small Leucine-rich Proteoglycans: From Genetics to Signal Transduction. J Biol Chem. 2008;283(31):21305–9. doi: 10.1074/jbc.R800020200 18463092 10.1074/jbc.R800020200 PMC2490788

[14] Merline R, Schaefer RM, Schaefer L. The matricellular functions of small leucine-rich proteoglycans (SLRPs). J Cell Commun Signal. 2009;3(3–4):323–35. doi: 10.1007/s12079-009-0066-2 19809894 10.1007/s12079-009-0066-2 PMC2778586

[15] Chen S, Birk DE. The regulatory roles of small leucine-rich proteoglycans in extracellular matrix assembly. FEBS J. 2013;280(10):2120–37. doi: 10.1111/febs.12136 23331954 10.1111/febs.12136 PMC3651807

[16] Schaefer L, Tredup C, Gubbiotti MA, Iozzo RV. Proteoglycan neofunctions: regulation of inflammation and autophagy in cancer biology. FEBS J. 2017 1;284(1):10–26. doi: 10.1111/febs.13963 27860287 10.1111/febs.13963 PMC5226885

[17] Theocharis AD, Skandalis SS, Neill T, Multhaupt HA, Hubo M, Frey H, et al Insights into the key roles of proteoglycans in breast cancer biology and translational medicine. Biochim Biophys Acta. 2015;1855(2):276–300. doi: 10.1016/j.bbcan.2015.03.006 25829250 10.1016/j.bbcan.2015.03.006 PMC4433619

[18] Neill T, Schaefer L, Iozzo RV. Oncosuppressive functions of decorin. Mol Cell Oncol. 2015;2(3):e975645 doi: 10.4161/23723556.2014.975645 27308453 10.4161/23723556.2014.975645 PMC4905288

[19] Karamanou K, Franchi M, Piperigkou Z, Perreau C, Maquart FX, Vynios DH, et al Lumican effectively regulates the estrogen receptors-associated functional properties of breast cancer cells, expression of matrix effectors and epithelial-to-mesenchymal transition. Sci Rep. 2017;7:45138 doi: 10.1038/srep45138 28332606 10.1038/srep45138 PMC5362815

[20] Kalamajski S, Oldberg A. The role of small leucine-rich proteoglycans in collagen fibrillogenesis. Matrix Biol. 2010;29(4):248–53. doi: 10.1016/j.matbio.2010.01.001 20080181 10.1016/j.matbio.2010.01.001

[21] Geng Y, McQuillan D, Roughley PJ. SLRP interaction can protect collagen fibrils from cleavage by collagenases. Matrix Biol. 2006;25(8):484–91. doi: 10.1016/j.matbio.2006.08.259 16979885 10.1016/j.matbio.2006.08.259

[22] Alimohamad H, Habijanac T, Larjava H, Häkkinen L. Colocalization of the collagen-binding proteoglycans decorin, biglycan, fibromodulin and lumican with different cells in human gingiva. J Periodontal Res. 2005;40(1):73–86. doi: 10.1111/j.1600-0765.2004.00776.x 15613083 10.1111/j.1600-0765.2004.00776.x

[23] Robinson KA, Sun M, Barnum CE, Weiss SN, Huegel J, Shetye SS, et al Decorin and biglycan are necessary for maintaining collagen fibril structure, fiber realignment, and mechanical properties of mature tendons. Matrix Biol. 2017;64:81–93. doi: 10.1016/j.matbio.2017.08.004 28882761 10.1016/j.matbio.2017.08.004 PMC5705405

[24] Ezura Y, Chakravarti S, Oldberg A, Chervoneva I, Birk DE. Differential expression of lumican and fibromodulin regulate collagen fibrillogenesis in developing mouse tendons. J Cell Biol. 2000;151(4):779–88. 11076963 10.1083/jcb.151.4.779 PMC2169450

[25] Kobayashi H, Higashiura Y, Shigetomi H, Kajihara H. Pathogenesis of endometriosis: the role of initial infection and subsequent sterile inflammation. Mol Med Rep. 2014;9(1):9–15. doi: 10.3892/mmr.2013.1755 24173432 10.3892/mmr.2013.1755

[26] DeLancey JO. Structural support of the urethra as it relates to stress urinary incontinence: the hammock hypothesis. Am J Obstet Gynecol. 1994;170(6):1713–20: discussion 1720–23. 8203431 10.1016/s0002-9378(94)70346-9

[27] Wen Y, Zhao YY, Li S, Polan ML, Chen BH. Differences in mRNA and protein expression of small proteoglycans in vaginal wall tissue from women with and without stress urinary incontinence. Hum Reprod. 2007;22(6):1718–24. doi: 10.1093/humrep/dem039 17395685 10.1093/humrep/dem039

[28] Nakatani T, Marui T, Hitora T, Doita M, Nishida K, Kurosaka M.Mechanical stretching force promotes collagen synthesis by cultured cells from human ligamentum flavum via transforming growth factor-beta1. J Orthop Res. 2002;20(6):1380–6. doi: 10.1016/S0736-0266(02)00046-3 12472256 10.1016/S0736-0266(02)00046-3

[29] Jia X, Huang R, Lei Z, Yao L, Wang L, Li Y, et al Detection of a novel large deletion causing α-thalassemia in South China. Experimental and Molecular Pathology. 2013;95(1):68–73. doi: 10.1016/j.yexmp.2013.05.007 23726795 10.1016/j.yexmp.2013.05.007

[30] Ruiz-Zapata AM, Kerkhof MH, Zandieh-Doulabi B, Brölmann HA, Smit TH, Helder MN. Fibroblasts from women with pelvic organ prolapse show differential mechanoresponses depending on surface substrates. Int Urogynecol J. 2013;24(9):1567–75. doi: 10.1007/s00192-013-2069-z 23579290 10.1007/s00192-013-2069-z PMC3745620

[31] Gautieri A, Vesentini S, Redaelli A, Buehler MJ. Viscoelastic properties of model segments of collagen molecules. Matrix Biol. 2012;31(2):141–9. doi: 10.1016/j.matbio.2011.11.005 22204879 10.1016/j.matbio.2011.11.005

[32] Ghodsi H, Darvish K. Investigation of mechanisms of viscoelastic behavior of collagen molecule. J Mech Behav Biomed Mater. 2015;51:194–204. doi: 10.1016/j.jmbbm.2015.07.015 26256473 10.1016/j.jmbbm.2015.07.015 PMC4581979

[33] Cowman MK, Schmidt TA, Raghavan P, Stecco A. Viscoelastic Properties of Hyaluronan in Physiological Conditions. F1000Res. 2015;4:622 doi: 10.12688/f1000research.6885.1 26594344 10.12688/f1000research.6885.1 PMC4648226

[34] Lelièvre SA. Contributions of extracellular matrix signaling and tissue architecture to nuclear mechanisms and spatial organization of gene expression control. Biochim Biophys Acta. 2009;1790(9):925–35. doi: 10.1016/j.bbagen.2009.03.013 19328836 10.1016/j.bbagen.2009.03.013 PMC2728154

[35] Chiquet M, Gelman L, Lutz R, Maier S. From mechanotransduction to extracellular matrix gene expression in fibroblasts. Biochim Biophys. Acta. 2009;1793(5):911–920. doi: 10.1016/j.bbamcr.2009.01.012 19339214 10.1016/j.bbamcr.2009.01.012

[36] Wang JH, Thampatty BP, Lin JS, Im HJ. Mechanoregulation of gene expression in fibroblasts. Gene 2007;391(1–2):1–15. doi: 10.1016/j.gene.2007.01.014 17331678 10.1016/j.gene.2007.01.014 PMC2893340

[37] Bildircin D, Kokcu A, Celik H, Sagir D, Kefeli M. Comparison of connective tissue components in the uterine ligaments between women with and without pelvic organ prolapse. Minerva Ginecol. 2014;66(2):201–8. 24848078

[38] Kim SG, Akaike T, Sasagaw T, Atomi Y, Kurosawa H. Gene expression of type I and type III collagen by mechanical stretch in anterior cruciate ligament cells. Cell Struct Funct. 2002;27(3):139–44. 12207044 10.1247/csf.27.139

[39] Yang G, Crawford RC, Wang JH. Proliferation and collagen production of human patellar tendon fibroblasts in response to cyclic uniaxial stretching in serum-free conditions. J Biomech. 2004;37(10):1543–50. doi: 10.1016/j.jbiomech.2004.01.005 15336929 10.1016/j.jbiomech.2004.01.005

[40] Kresse H, Hausser H, Schönherr E. Small proteoglycans. Experientia. 1993;49(5):403–16. 8500596 10.1007/BF01923585

[41] Schönherr E, Witsch-Prehm P, Harrach B, Robenek H, Rauterberg J, Kresse H. Interaction of biglycan with type I collagen. J Biol Chem. 1995;270(6):2776–83. 7852349 10.1074/jbc.270.6.2776

[42] Svensson L, Heinegård D, Oldberg A. Decorin-binding sites for collagen type I are mainly located in leucine-rich repeats 4–5. J Biol Chem. 1995;270(35):20712–6. 7657652 10.1074/jbc.270.35.20712

[43] Kalamajski S, Oldberg A. Homologous sequence in lumican and fibromodulin leucine-rich repeat 5–7 competes for collagen binding. J Biol Chem. 2009;284(1):534–9. doi: 10.1074/jbc.M805721200 19008226 10.1074/jbc.M805721200

[44] Svensson L, Närlid I, Oldberg A. Fibromodulin and lumican bind to the same region on collagen type I fibrils. FEBS Lett. 2000;470(2):178–82. 10734230 10.1016/s0014-5793(00)01314-4

[45] Neame PJ, Kay CJ, McQuillan DJ, Beales MP, Hassell JR. Independent modulation of collagen fibrillogenesis by decorin and lumican. Cell Mol Life Sci. 2000;57(5):859–63. doi: 10.1007/s000180050048 10892350 10.1007/s000180050048 PMC11146782

[46] Hedbom E, Heinegård D. Binding of fibromodulin and decorin to separate sites on fibrillar collagens. J Biol Chem. 1993;268(36):27307–12. 8262971

[47] Scott JE. Proteoglycan-fibrillar collagen interactions. Biochem J. 1988;252(2):313–23. 3046606 10.1042/bj2520313 PMC1149146

[48] Scott PG, McEwan PA, Dodd CM, Bergmann EM, Bishop PN, Bella J. Crystal structure of the dimeric protein core of decorin, the archetypal small leucine-rich repeat proteoglycan. Proc Natl Acad Sci U S A. 2004;101(44):15633–8. doi: 10.1073/pnas.0402976101 15501918 10.1073/pnas.0402976101 PMC524833

[49] Zhang G, Ezura Y, Chervoneva I, Robinson PS, Beason DP, Carine ET, et al Decorin regulates assembly of collagen fibrils and acquisition of biomechanical properties during tendon development. J Cell Biochem. 2006;98(6):1436–49. doi: 10.1002/jcb.20776 16518859 10.1002/jcb.20776

[50] Zhang G, Chen S, Goldoni S, Calder BW, Simpson HC, Owens RT, et al Genetic evidence for the coordinated regulation of collagen fibrillogenesis in the cornea by decorin and biglycan. J Biol Chem. 2009;284(13):8888–97. doi: 10.1074/jbc.M806590200 19136671 10.1074/jbc.M806590200 PMC2659246

[51] Ezura Y, Chakravarti S, Oldberg A, Chervoneva I, Birk DE. Differential expression of lumican and fibromodulin regulate collagen fibrillogenesis in developing mouse tendons. J Cell Biol. 2000;151(4):779–88. 11076963 10.1083/jcb.151.4.779 PMC2169450

[52] Jepsen KJ, Wu F, Peragallo JH, Paul J, Roberts L, Ezura Y, et al A syndrome of joint laxity and impaired tendon integrity in lumican- and fibromodulin-deficient mice. J Biol Chem. 2002;277(38):35532–40. doi: 10.1074/jbc.M205398200 12089156 10.1074/jbc.M205398200

[53] Ewies AA, Elshafie M, Li J, Stanley A, Thompson J, Styles J, et al Changes in transcription profile and cytoskeleton morphology in pelvic ligament fibroblasts in response to stretch: The effects of estradiol and levormeloxifene. Mol HumReprod. 2008; 4(2):127–35. doi: 10.1093/molehr/gam090 18184756 10.1093/molehr/gam090

[54] Liu YM, Choy KW, Lui WT, Pang MW, Wong YF, Yip SK. 17 β-estradiol suppresses proliferation of fibroblasts derived from cardinal ligaments in patients with or without pelvic organ prolapse. Hum Reprod. 2006;21(1):303–8. doi: 10.1093/humrep/dei296 16155073 10.1093/humrep/dei296

[55] Markiewicz M, Znoyko S, Stawski L, Ghatnekar A, Gilkeson G, Trojanowska M. A role for estrogen receptor-α and estrogen receptor-β in collagen biosynthesis in mouse skin. J Invest Dermatol. 2013;133(1):120–7. doi: 10.1038/jid.2012.264 22895361 10.1038/jid.2012.264 PMC3502697

[56] Jin L, Zhang XH, Wang JL, Yu YZ. Expression of estrogen receptor alpha and beta subtypes in the vaginal wall of women with anterior vaginal prolapse]. Zhonghua Fu Chan Ke Za Zhi. 2007;42(1):18–21. [Article in Chinese] 17331415

[57] Fuermetz A, Schoenfeld M, Ennemoser S, Muetzel E, Jeschke U, Jundt K. Change of steroid receptor expression in the posterior vaginal wall after local estrogen therapy. Eur J Obstet Gynecol Reprod Biol. 2015;187:45–50. doi: 10.1016/j.ejogrb.2015.02.021 25748487 10.1016/j.ejogrb.2015.02.021

